# 2-(1*H*-Benzotriazol-1-yl)-3-(2,6-dichloro­phen­yl)-1-phenyl­propan-1-ol

**DOI:** 10.1107/S1600536811034738

**Published:** 2011-08-27

**Authors:** Özden Özel Güven, Seval Çapanlar, Simon J. Coles, Tuncer Hökelek

**Affiliations:** a Department of Chemistry, Zonguldak Karaelmas University, 67100 Zonguldak, Turkey; b Department of Chemistry, Southampton University, SO17 1BJ Southampton, England; c Department of Physics, Hacettepe University, 06800 Beytepe, Ankara, Turkey

## Abstract

The asymmetric unit of the title compound, C_21_H_17_Cl_2_N_3_O, contains two crystallographically independent mol­ecules with similar conformations. The benzotriazole ring is oriented at dihedral angles of 30.61 (5) and 43.36 (5)°, respectively, to the phenyl and dichloro­phenyl rings in one mol­ecule, and 32.25 (5) and 41.04 (5)° in the other. The dihedral angles between the phenyl and dichloro­phenyl rings are 66.38 (7) and 66.14 (6)° in the two mol­ecules. An intra­molecular O—H⋯N hydrogen bond links the benzotriazole ring and phenyl­propanol unit in each mol­ecule. In the crystal, weak inter­molecular C—H⋯N hydrogen bonds link the mol­ecules into chains along the *a* axis. π–π stacking between the dichloro­phenyl rings [centroid–centroid distances = 3.809 (1) and 3.735 (1) Å] may further stabilize the crystal structure.

## Related literature

For the biological activity of azole compounds, see: Cozzi *et al.* (1994 ▶) and of triazole derivatives, see: Jin *et al.* (2006 ▶). For related structures, see: Özel Güven *et al.* (2007 ▶, 2008 ▶, 2010 ▶).
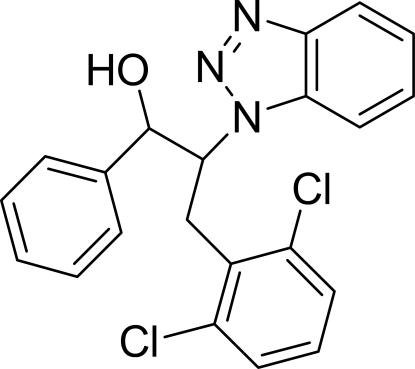


## Experimental

### 

#### Crystal data

C_21_H_17_Cl_2_N_3_O
*M* *_r_* = 398.28
Triclinic, 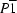

*a* = 9.3894 (2) Å
*b* = 9.4947 (2) Å
*c* = 21.2687 (3) Å
α = 91.415 (2)°
β = 92.324 (2)°
γ = 90.406 (1)°
*V* = 1893.90 (6) Å^3^
*Z* = 4
Mo *K*α radiation
μ = 0.36 mm^−1^
*T* = 120 K
0.4 × 0.4 × 0.3 mm

#### Data collection

Nonius KappaCCD diffractometer
Absorption correction: multi-scan (*SADABS*; Sheldrick, 2007 ▶) *T*_min_ = 0.866, *T*_max_ = 0.897
40396 measured reflections
8697 independent reflections
7206 reflections with *I* > 2σ(*I*)
*R*_int_ = 0.044

#### Refinement

*R*[*F* ^2^ > 2σ(*F* ^2^)] = 0.042
*wR*(*F* ^2^) = 0.108
*S* = 1.07
8697 reflections
487 parameters
H-atom parameters constrained
Δρ_max_ = 0.43 e Å^−3^
Δρ_min_ = −0.73 e Å^−3^

### 

Data collection: *COLLECT* (Nonius, 1998 ▶); cell refinement: *DENZO* (Otwinowski & Minor, 1997 ▶) and *COLLECT*; data reduction: *DENZO* and *COLLECT*; program(s) used to solve structure: *SHELXS97* (Sheldrick, 2008 ▶); program(s) used to refine structure: *SHELXL97* (Sheldrick, 2008 ▶); molecular graphics: *ORTEP-3 for Windows* (Farrugia, 1997 ▶); software used to prepare material for publication: *WinGX* (Farrugia, 1999 ▶) and *PLATON* (Spek, 2009 ▶).

## Supplementary Material

Crystal structure: contains datablock(s) I, global. DOI: 10.1107/S1600536811034738/xu5297sup1.cif

Structure factors: contains datablock(s) I. DOI: 10.1107/S1600536811034738/xu5297Isup2.hkl

Supplementary material file. DOI: 10.1107/S1600536811034738/xu5297Isup3.cml

Additional supplementary materials:  crystallographic information; 3D view; checkCIF report

## Figures and Tables

**Table 1:** Hydrogen-bond geometry (Å, °)

*D*—H⋯*A*	*D*—H	H⋯*A*	*D*⋯*A*	*D*—H⋯*A*
O1—H1*A*⋯N2	0.82	2.56	3.1254 (19)	127
O1′—H1*B*⋯N2′	0.82	2.52	3.0881 (19)	128
C19—H19⋯N3^i^	0.93	2.54	3.420 (2)	158
C19′—H19′⋯N3′^ii^	0.93	2.53	3.410 (2)	159

Symmetry codes: (i) 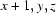; (ii) 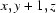.

**Fig. 1.**
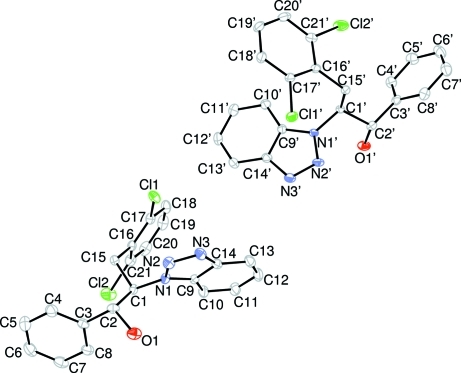
The molecular structure of the title molecule with the atom-numbering scheme. Displacement ellipsoids are drawn at the 50% probability level. H-atoms have been omitted for clarity.

**Fig. 2.**
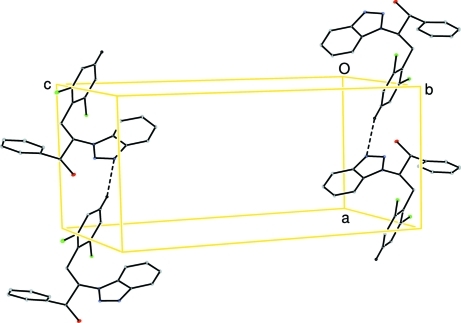
A view of the crystal packing of the title compound. The intermolecular C—H···N hydrogen bonds are shown as dashed lines [H-atoms not involved in hydrogen bonding have been omitted for clarity].

## supplementary crystallographic information

### Comment

Azole compounds have important biological activities. In literature 2-(1*H*-imidazol-1-yl)-1,3-diphenylpropan-1-one and its derivatives have been reported that they show both high and selective thromboxane A2 receptor antagonist and thromboxane A2 synthase inhibitory activity (Cozzi *et al.*, 1994). Some 1*H*-1,2,4-triazole derivatives have been known as antifungal and plant growth regulatory agents (Jin *et al.*, 2006). Crystal structures of similar compounds like ketone having benzimidazole and furan rings (Özel Güven *et al.*, 2007) and alcohols having 1,2,4-triazole and benzotriazole rings have been reported (Özel Güven *et al.*, 2008; Özel Güven *et al.*, 2010). Now, we report herein the crystal structure of the title benzotriazole derivative, (I).

The asymmetric unit of the title compound (Fig. 1) contains two crystallographically independent molecules, in which they differ slightly in the orientations of the 2-6-dichlorophenyl units and the bond lengths and angles are generally within normal ranges. The intramolecular O—H···N hydrogen bonds (Table 1) link the benzotriazole rings and phenylpropan units. In each molecule, the planar benzotriazole rings [A (N1-N3/C9-C14) and A' (N1'-N3'/C9'-C14')] are oriented with respect to the phenyl [B (C3-C8) and B' (C3'-C8')] and 2-6-dichlorophenyl [C (C16-C21)] and C' (C16'-C21')] rings at dihedral angles of A/B = 30.61 (5), A/C = 43.36 (5) ° and A'/B' = 32.25 (5), A'/C' = 41.04 (5) °. The dihedral angles between phenyl rings are B/C = 66.38 (7) and B'/C' = 66.14 (6) °. Atoms C1 and C1' are -0.044 (2) and 0.086 (2) Å away from the planes of the benzotriazole rings, respectively.

In the crystal structure, intermolecular C—H···N hydrogen bonds (Table 1) link the molecules into chains along the a-axis (Fig. 2). The π–π contacts between the 2-6-dichlorophenyl rings, Cg3—Cg3^i^ and Cg3'—Cg3'^ii^, [symmetry codes: (i) 2 - x, 2 - y, - z, (ii) 1 - x, 1 - y, 1 - z, where Cg3 and Cg3' are the centroids of the rings C (C16-C21)] and C' (C16'-C21'), respectively], may further stabilize the structure, with centroid-centroid distances of 3.809 (1) and 3.735 (1) Å, respectively.

### Experimental

The title compound, (I), was synthesized by the reduction of 2-(1*H*-benzotriazol-1-yl)-3-(2,6-dichlorophenyl)-1-phenylpropane-1-one. Sodiumborohydride (38.21 mg, 1.01 mmol) was added to a solution of 2-(1*H*-benzotriazol-1-yl)-3-(2,6-dichlorophenyl)-1-phenylpropane-1-one (200 mg, 0.505 mmol) in ethanol (15 ml). The mixture was refluxed for 5 h. The solvent was removed and the mixture was neutralized with dilute HCl, and then refluxed for 30 min. After cooling the mixture, it was alkalinized with dilute NaOH. The precipitate was filtered and washed with ethanol. The filtrate was extracted with chloroform, then the organic phase was dried and solvents were removed by rotary evaporator. The residue was purified with column chromatography using hexane: ethyl acetate (7:3) mixture as solvent. The product was crystallized from benzene to obtain colorless single crystals suitable for X-ray analysis (yield; 82 mg, 41%).

### Refinement

H atoms were positioned geometrically with O—H = 0.82 for hydroxy H, and C—H = 0.93, 0.97 and 0.98 Å for aromatic, methylene and methine H-atoms, respectively, and constrained to ride on their parent atoms, with *U*_iso_(H) = k × *U*_eq_(C,O), where k = 1.5 for hydroxy H-atoms and k = 1.2 for all other H-atoms.

### Crystal data

**Table:**

C_21_H_17_Cl_2_N_3_O	*Z* = 4
*M**_r_* = 398.28	*F*(000) = 824
Triclinic, *P*1	*D*_x_ = 1.397 Mg m^−^^3^
Hall symbol: -P 1	Mo *K*α radiation, λ = 0.71073 Å
*a* = 9.3894 (2) Å	Cell parameters from 14651 reflections
*b* = 9.4947 (2) Å	θ = 2.9–27.5°
*c* = 21.2687 (3) Å	µ = 0.36 mm^−^^1^
α = 91.415 (2)°	*T* = 120 K
β = 92.324 (2)°	Block, colorless
γ = 90.406 (1)°	0.4 × 0.4 × 0.3 mm
*V* = 1893.90 (6) Å^3^	

### Data collection

**Table:**

Nonius KappaCCD diffractometer	8697 independent reflections
Radiation source: fine-focus sealed tube	7206 reflections with *I* > 2σ(*I*)
graphite	*R*_int_ = 0.044
φ and ω scans	θ_max_ = 27.6°, θ_min_ = 3.0°
Absorption correction: multi-scan (*SADABS*; Sheldrick, 2007)	*h* = −12→12
*T*_min_ = 0.866, *T*_max_ = 0.897	*k* = −12→11
40396 measured reflections	*l* = −27→27

### Refinement

**Table:**

Refinement on *F*^2^	Primary atom site location: structure-invariant direct methods
Least-squares matrix: full	Secondary atom site location: difference Fourier map
*R*[*F*^2^ > 2σ(*F*^2^)] = 0.042	Hydrogen site location: inferred from neighbouring sites
*wR*(*F*^2^) = 0.108	H-atom parameters constrained
*S* = 1.07	*w* = 1/[σ^2^(*F*_o_^2^) + (0.0504*P*)^2^ + 0.9534*P*] where *P* = (*F*_o_^2^ + 2*F*_c_^2^)/3
8697 reflections	(Δ/σ)_max_ = 0.001
487 parameters	Δρ_max_ = 0.43 e Å^−^^3^
0 restraints	Δρ_min_ = −0.73 e Å^−^^3^

### Special details

**Table:**

Geometry. All esds (except the esd in the dihedral angle between two l.s. planes) are estimated using the full covariance matrix. The cell esds are taken into account individually in the estimation of esds in distances, angles and torsion angles; correlations between esds in cell parameters are only used when they are defined by crystal symmetry. An approximate (isotropic) treatment of cell esds is used for estimating esds involving l.s. planes.
Refinement. Refinement of F^2^ against ALL reflections. The weighted R-factor wR and goodness of fit S are based on F^2^, conventional R-factors R are based on F, with F set to zero for negative F^2^. The threshold expression of F^2^ > 2sigma(F^2^) is used only for calculating R-factors(gt) etc. and is not relevant to the choice of reflections for refinement. R-factors based on F^2^ are statistically about twice as large as those based on F, and R- factors based on ALL data will be even larger.

### Fractional atomic coordinates and isotropic or equivalent isotropic displacement parameters (Å^2^)

**Table:**

	*x*	*y*	*z*	*U*_iso_*/*U*_eq_	
Cl1	0.77473 (4)	1.06219 (5)	0.104106 (19)	0.02207 (10)	
Cl2	0.97499 (5)	0.66618 (5)	−0.05798 (2)	0.03245 (12)	
O1	0.42613 (13)	0.61760 (13)	−0.02537 (6)	0.0256 (3)	
H1A	0.4009	0.6380	0.0101	0.038*	
N1	0.60715 (15)	0.74669 (14)	0.06575 (6)	0.0162 (3)	
N2	0.50172 (15)	0.83379 (15)	0.08432 (7)	0.0200 (3)	
N3	0.49164 (16)	0.82583 (16)	0.14548 (7)	0.0206 (3)	
C1	0.64956 (17)	0.73778 (17)	0.00021 (7)	0.0149 (3)	
H1	0.7061	0.6522	−0.0048	0.018*	
C2	0.52030 (18)	0.72524 (18)	−0.04605 (8)	0.0193 (3)	
H2	0.4698	0.8152	−0.0462	0.023*	
C3	0.57040 (18)	0.69279 (18)	−0.11180 (8)	0.0197 (4)	
C4	0.5683 (2)	0.7957 (2)	−0.15719 (9)	0.0284 (4)	
H4	0.5318	0.8842	−0.1479	0.034*	
C5	0.6205 (3)	0.7673 (2)	−0.21634 (10)	0.0366 (5)	
H5	0.6183	0.8366	−0.2465	0.044*	
C6	0.6758 (2)	0.6356 (2)	−0.23049 (10)	0.0338 (5)	
H6	0.7117	0.6171	−0.2699	0.041*	
C7	0.6774 (2)	0.5318 (2)	−0.18587 (9)	0.0295 (4)	
H7	0.7136	0.4433	−0.1955	0.035*	
C8	0.6248 (2)	0.55996 (19)	−0.12650 (9)	0.0237 (4)	
H8	0.6260	0.4901	−0.0966	0.028*	
C9	0.66758 (18)	0.68088 (17)	0.11643 (8)	0.0160 (3)	
C10	0.7773 (2)	0.58173 (18)	0.12292 (8)	0.0215 (4)	
H10	0.8286	0.5502	0.0889	0.026*	
C11	0.8040 (2)	0.5342 (2)	0.18290 (9)	0.0258 (4)	
H11	0.8748	0.4676	0.1894	0.031*	
C12	0.7273 (2)	0.5834 (2)	0.23490 (9)	0.0262 (4)	
H12	0.7481	0.5472	0.2744	0.031*	
C13	0.6226 (2)	0.6834 (2)	0.22842 (8)	0.0237 (4)	
H13	0.5740	0.7172	0.2628	0.028*	
C14	0.59246 (18)	0.73200 (17)	0.16758 (8)	0.0173 (3)	
C15	0.74540 (17)	0.86394 (17)	−0.01401 (8)	0.0157 (3)	
H15A	0.7664	0.8609	−0.0583	0.019*	
H15B	0.6948	0.9506	−0.0055	0.019*	
C16	0.88295 (17)	0.86400 (17)	0.02488 (8)	0.0166 (3)	
C17	0.90693 (18)	0.94709 (18)	0.07957 (8)	0.0183 (3)	
C18	1.03235 (19)	0.9434 (2)	0.11631 (9)	0.0238 (4)	
H18	1.0437	0.9998	0.1526	0.029*	
C19	1.1407 (2)	0.8542 (2)	0.09808 (9)	0.0289 (4)	
H19	1.2255	0.8510	0.1222	0.035*	
C20	1.12324 (19)	0.7704 (2)	0.04434 (10)	0.0276 (4)	
H20	1.1960	0.7113	0.0319	0.033*	
C21	0.99607 (19)	0.77537 (19)	0.00931 (9)	0.0225 (4)	
Cl1'	0.43627 (5)	1.25631 (4)	0.394633 (19)	0.02133 (10)	
Cl2'	0.83607 (5)	1.47909 (5)	0.56172 (2)	0.02874 (12)	
O1'	0.88141 (13)	0.93042 (12)	0.52629 (6)	0.0217 (3)	
H1B	0.8569	0.8991	0.4912	0.032*	
N1'	0.74653 (15)	1.09289 (14)	0.43580 (6)	0.0143 (3)	
N2'	0.65658 (15)	0.98414 (14)	0.41974 (7)	0.0184 (3)	
N3'	0.65629 (16)	0.96282 (15)	0.35860 (7)	0.0201 (3)	
C1'	0.76151 (17)	1.14764 (16)	0.50055 (7)	0.0134 (3)	
H1'	0.8488	1.2049	0.5043	0.016*	
C2'	0.77665 (18)	1.02758 (17)	0.54764 (8)	0.0163 (3)	
H2'	0.6849	0.9782	0.5494	0.020*	
C3'	0.81778 (18)	1.08827 (17)	0.61265 (8)	0.0167 (3)	
C4'	0.95523 (19)	1.14282 (18)	0.62497 (8)	0.0207 (4)	
H4'	1.0218	1.1385	0.5938	0.025*	
C5'	0.9927 (2)	1.2033 (2)	0.68340 (9)	0.0251 (4)	
H5'	1.0840	1.2402	0.6911	0.030*	
C6'	0.8942 (2)	1.2090 (2)	0.73039 (9)	0.0283 (4)	
H6'	0.9194	1.2499	0.7695	0.034*	
C7'	0.7588 (2)	1.1537 (2)	0.71895 (9)	0.0278 (4)	
H7'	0.6932	1.1569	0.7505	0.033*	
C8'	0.7202 (2)	1.09329 (19)	0.66023 (8)	0.0224 (4)	
H8'	0.6289	1.0562	0.6528	0.027*	
C9'	0.80557 (17)	1.14485 (17)	0.38334 (7)	0.0150 (3)	
C10'	0.90362 (19)	1.25377 (18)	0.37405 (8)	0.0200 (4)	
H10'	0.9412	1.3100	0.4072	0.024*	
C11'	0.9407 (2)	1.2722 (2)	0.31273 (9)	0.0272 (4)	
H11'	1.0059	1.3427	0.3042	0.033*	
C12'	0.8824 (2)	1.1869 (2)	0.26232 (9)	0.0309 (4)	
H12'	0.9107	1.2029	0.2217	0.037*	
C13'	0.7850 (2)	1.08096 (19)	0.27169 (8)	0.0262 (4)	
H13'	0.7465	1.0257	0.2384	0.031*	
C14'	0.74682 (19)	1.06059 (17)	0.33400 (8)	0.0180 (3)	
C15'	0.63528 (17)	1.24411 (16)	0.51530 (7)	0.0146 (3)	
H15C	0.5466	1.1929	0.5066	0.017*	
H15D	0.6403	1.2706	0.5597	0.017*	
C16'	0.63594 (18)	1.37529 (16)	0.47679 (8)	0.0160 (3)	
C17'	0.55197 (18)	1.39105 (17)	0.42163 (8)	0.0167 (3)	
C18'	0.5560 (2)	1.51185 (18)	0.38579 (8)	0.0213 (4)	
H18'	0.4990	1.5184	0.3492	0.026*	
C19'	0.6460 (2)	1.62216 (18)	0.40542 (9)	0.0237 (4)	
H19'	0.6494	1.7032	0.3818	0.028*	
C20'	0.7307 (2)	1.61274 (18)	0.45971 (9)	0.0238 (4)	
H20'	0.7903	1.6871	0.4731	0.029*	
C21'	0.72545 (19)	1.49006 (18)	0.49406 (8)	0.0193 (3)	

### Atomic displacement parameters (Å^2^)

**Table:**

	*U*^11^	*U*^22^	*U*^33^	*U*^12^	*U*^13^	*U*^23^
Cl1	0.0183 (2)	0.0301 (2)	0.0176 (2)	0.00322 (16)	0.00066 (16)	−0.00500 (16)
Cl2	0.0273 (2)	0.0317 (3)	0.0388 (3)	0.00458 (19)	0.0124 (2)	−0.0105 (2)
O1	0.0194 (6)	0.0270 (7)	0.0307 (7)	−0.0052 (5)	0.0077 (5)	−0.0054 (5)
N1	0.0161 (7)	0.0160 (7)	0.0170 (7)	0.0044 (5)	0.0052 (5)	0.0029 (5)
N2	0.0157 (7)	0.0215 (7)	0.0233 (8)	0.0046 (6)	0.0058 (6)	−0.0001 (6)
N3	0.0177 (7)	0.0252 (8)	0.0190 (7)	0.0010 (6)	0.0038 (6)	−0.0031 (6)
C1	0.0155 (8)	0.0155 (8)	0.0139 (8)	0.0033 (6)	0.0034 (6)	0.0018 (6)
C2	0.0154 (8)	0.0177 (8)	0.0244 (9)	−0.0001 (6)	0.0000 (7)	−0.0025 (7)
C3	0.0174 (8)	0.0211 (8)	0.0202 (9)	−0.0008 (7)	−0.0030 (7)	−0.0031 (7)
C4	0.0334 (11)	0.0256 (10)	0.0256 (10)	0.0024 (8)	−0.0051 (8)	−0.0002 (8)
C5	0.0459 (13)	0.0398 (12)	0.0240 (10)	−0.0039 (10)	−0.0013 (9)	0.0067 (9)
C6	0.0345 (11)	0.0463 (12)	0.0205 (10)	−0.0060 (9)	0.0039 (8)	−0.0059 (8)
C7	0.0299 (11)	0.0288 (10)	0.0295 (10)	−0.0005 (8)	0.0044 (8)	−0.0115 (8)
C8	0.0251 (9)	0.0219 (9)	0.0239 (9)	−0.0022 (7)	0.0029 (7)	−0.0040 (7)
C9	0.0177 (8)	0.0145 (8)	0.0158 (8)	−0.0008 (6)	0.0018 (6)	0.0006 (6)
C10	0.0256 (9)	0.0210 (9)	0.0183 (8)	0.0065 (7)	0.0037 (7)	0.0012 (7)
C11	0.0294 (10)	0.0244 (9)	0.0238 (9)	0.0067 (8)	−0.0019 (8)	0.0052 (7)
C12	0.0298 (10)	0.0321 (10)	0.0167 (9)	−0.0015 (8)	−0.0021 (7)	0.0052 (7)
C13	0.0231 (9)	0.0308 (10)	0.0170 (8)	−0.0040 (7)	0.0038 (7)	−0.0035 (7)
C14	0.0150 (8)	0.0186 (8)	0.0184 (8)	−0.0027 (6)	0.0029 (6)	−0.0023 (6)
C15	0.0155 (8)	0.0176 (8)	0.0140 (8)	0.0000 (6)	0.0010 (6)	0.0005 (6)
C16	0.0139 (8)	0.0198 (8)	0.0165 (8)	−0.0003 (6)	0.0028 (6)	0.0038 (6)
C17	0.0134 (8)	0.0232 (9)	0.0185 (8)	−0.0002 (6)	0.0032 (6)	0.0040 (7)
C18	0.0175 (9)	0.0347 (10)	0.0191 (9)	−0.0029 (7)	−0.0022 (7)	0.0059 (7)
C19	0.0139 (9)	0.0434 (12)	0.0298 (10)	0.0001 (8)	−0.0012 (7)	0.0144 (9)
C20	0.0148 (9)	0.0320 (10)	0.0373 (11)	0.0061 (7)	0.0081 (8)	0.0102 (8)
C21	0.0174 (9)	0.0256 (9)	0.0250 (9)	0.0005 (7)	0.0072 (7)	0.0028 (7)
Cl1'	0.0268 (2)	0.0159 (2)	0.0207 (2)	−0.00349 (16)	−0.00538 (17)	0.00179 (15)
Cl2'	0.0305 (3)	0.0212 (2)	0.0332 (3)	−0.00356 (17)	−0.01093 (19)	−0.00507 (18)
O1'	0.0232 (6)	0.0154 (6)	0.0258 (7)	0.0046 (5)	−0.0037 (5)	−0.0032 (5)
N1'	0.0161 (7)	0.0123 (6)	0.0146 (7)	−0.0029 (5)	0.0016 (5)	−0.0021 (5)
N2'	0.0200 (7)	0.0137 (7)	0.0211 (7)	−0.0041 (5)	−0.0005 (6)	−0.0042 (5)
N3'	0.0253 (8)	0.0154 (7)	0.0189 (7)	−0.0010 (6)	−0.0030 (6)	−0.0025 (5)
C1'	0.0147 (8)	0.0121 (7)	0.0133 (7)	−0.0023 (6)	0.0008 (6)	−0.0014 (6)
C2'	0.0158 (8)	0.0136 (8)	0.0193 (8)	−0.0012 (6)	−0.0009 (6)	0.0027 (6)
C3'	0.0186 (8)	0.0140 (8)	0.0173 (8)	0.0019 (6)	−0.0024 (6)	0.0040 (6)
C4'	0.0184 (9)	0.0237 (9)	0.0200 (9)	0.0011 (7)	−0.0011 (7)	0.0009 (7)
C5'	0.0218 (9)	0.0266 (9)	0.0262 (10)	−0.0010 (7)	−0.0061 (7)	−0.0016 (7)
C6'	0.0340 (11)	0.0314 (10)	0.0189 (9)	0.0071 (8)	−0.0046 (8)	−0.0028 (7)
C7'	0.0307 (10)	0.0322 (10)	0.0210 (9)	0.0047 (8)	0.0052 (8)	0.0021 (8)
C8'	0.0194 (9)	0.0243 (9)	0.0236 (9)	0.0008 (7)	0.0014 (7)	0.0046 (7)
C9'	0.0152 (8)	0.0149 (8)	0.0148 (8)	0.0025 (6)	0.0014 (6)	0.0005 (6)
C10'	0.0203 (9)	0.0204 (8)	0.0192 (8)	−0.0046 (7)	0.0017 (7)	−0.0008 (6)
C11'	0.0325 (11)	0.0261 (10)	0.0235 (9)	−0.0057 (8)	0.0074 (8)	0.0045 (7)
C12'	0.0500 (13)	0.0278 (10)	0.0157 (9)	0.0001 (9)	0.0088 (8)	0.0034 (7)
C13'	0.0429 (12)	0.0204 (9)	0.0148 (8)	0.0000 (8)	−0.0025 (8)	−0.0017 (7)
C14'	0.0217 (9)	0.0145 (8)	0.0177 (8)	0.0025 (6)	−0.0018 (7)	−0.0005 (6)
C15'	0.0170 (8)	0.0129 (7)	0.0140 (8)	0.0005 (6)	0.0022 (6)	0.0007 (6)
C16'	0.0199 (8)	0.0114 (7)	0.0170 (8)	0.0022 (6)	0.0045 (6)	−0.0008 (6)
C17'	0.0207 (8)	0.0125 (7)	0.0170 (8)	0.0000 (6)	0.0041 (6)	−0.0009 (6)
C18'	0.0289 (10)	0.0178 (8)	0.0176 (8)	0.0037 (7)	0.0051 (7)	0.0033 (6)
C19'	0.0334 (10)	0.0126 (8)	0.0259 (9)	0.0000 (7)	0.0105 (8)	0.0040 (7)
C20'	0.0272 (10)	0.0125 (8)	0.0321 (10)	−0.0037 (7)	0.0082 (8)	−0.0026 (7)
C21'	0.0201 (9)	0.0165 (8)	0.0212 (9)	0.0003 (6)	0.0012 (7)	−0.0036 (6)

### Geometric parameters (Å, °)

**Table:**

Cl1—C17	1.7467 (17)	Cl1'—C17'	1.7435 (17)
Cl2—C21	1.7500 (19)	Cl2'—C21'	1.7460 (18)
O1—C2	1.435 (2)	O1'—C2'	1.433 (2)
O1—H1A	0.8200	O1'—H1B	0.8200
N1—N2	1.3591 (19)	N1'—C1'	1.461 (2)
N1—C1	1.466 (2)	N1'—C9'	1.367 (2)
N1—C9	1.365 (2)	N2'—N1'	1.3576 (19)
N2—N3	1.312 (2)	N2'—N3'	1.311 (2)
N3—C14	1.381 (2)	N3'—C14'	1.381 (2)
C1—C2	1.533 (2)	C1'—C2'	1.540 (2)
C1—C15	1.536 (2)	C1'—C15'	1.541 (2)
C1—H1	0.9800	C1'—H1'	0.9800
C2—H2	0.9800	C2'—C3'	1.519 (2)
C3—C2	1.519 (2)	C2'—H2'	0.9800
C3—C4	1.390 (3)	C3'—C4'	1.400 (2)
C3—C8	1.397 (2)	C3'—C8'	1.393 (2)
C4—C5	1.389 (3)	C4'—C5'	1.387 (3)
C4—H4	0.9300	C4'—H4'	0.9300
C5—H5	0.9300	C5'—H5'	0.9300
C6—C5	1.388 (3)	C6'—C7'	1.382 (3)
C6—H6	0.9300	C6'—C5'	1.389 (3)
C7—C6	1.385 (3)	C6'—H6'	0.9300
C7—H7	0.9300	C7'—H7'	0.9300
C8—C7	1.395 (3)	C8'—C7'	1.395 (3)
C8—H8	0.9300	C8'—H8'	0.9300
C9—C10	1.405 (2)	C9'—C10'	1.403 (2)
C9—C14	1.399 (2)	C9'—C14'	1.397 (2)
C10—C11	1.378 (3)	C10'—C11'	1.379 (2)
C10—H10	0.9300	C10'—H10'	0.9300
C11—H11	0.9300	C11'—C12'	1.418 (3)
C12—C11	1.415 (3)	C11'—H11'	0.9300
C12—H12	0.9300	C12'—H12'	0.9300
C13—C12	1.377 (3)	C13'—C12'	1.378 (3)
C13—C14	1.403 (2)	C13'—H13'	0.9300
C13—H13	0.9300	C14'—C13'	1.404 (2)
C15—H15A	0.9700	C15'—H15C	0.9700
C15—H15B	0.9700	C15'—H15D	0.9700
C16—C15	1.505 (2)	C16'—C15'	1.508 (2)
C16—C17	1.399 (2)	C16'—C17'	1.398 (2)
C16—C21	1.405 (2)	C16'—C21'	1.404 (2)
C17—C18	1.388 (2)	C17'—C18'	1.394 (2)
C18—C19	1.390 (3)	C18'—C19'	1.387 (3)
C18—H18	0.9300	C18'—H18'	0.9300
C19—H19	0.9300	C19'—H19'	0.9300
C20—C19	1.380 (3)	C20'—C19'	1.380 (3)
C20—H20	0.9300	C20'—C21'	1.392 (2)
C21—C20	1.383 (3)	C20'—H20'	0.9300
			
C2—O1—H1A	109.5	C2'—O1'—H1B	109.5
N2—N1—C1	121.83 (13)	N2'—N1'—C1'	121.39 (13)
N2—N1—C9	110.25 (13)	N2'—N1'—C9'	110.31 (13)
C9—N1—C1	127.81 (13)	C9'—N1'—C1'	128.08 (13)
N3—N2—N1	108.75 (13)	N3'—N2'—N1'	108.85 (13)
N2—N3—C14	108.35 (13)	N2'—N3'—C14'	108.13 (13)
N1—C1—C2	111.92 (13)	N1'—C1'—C2'	111.37 (12)
N1—C1—C15	110.06 (13)	N1'—C1'—C15'	110.12 (13)
N1—C1—H1	107.6	N1'—C1'—H1'	107.8
C2—C1—C15	111.85 (13)	C2'—C1'—C15'	111.68 (13)
C2—C1—H1	107.6	C2'—C1'—H1'	107.8
C15—C1—H1	107.6	C15'—C1'—H1'	107.8
O1—C2—C3	111.67 (14)	O1'—C2'—C3'	111.31 (13)
O1—C2—C1	108.97 (14)	O1'—C2'—C1'	109.00 (13)
O1—C2—H2	108.9	O1'—C2'—H2'	108.9
C1—C2—H2	108.9	C1'—C2'—H2'	108.9
C3—C2—C1	109.52 (13)	C3'—C2'—C1'	109.66 (13)
C3—C2—H2	108.9	C3'—C2'—H2'	108.9
C4—C3—C2	120.78 (16)	C4'—C3'—C2'	119.74 (15)
C4—C3—C8	119.18 (17)	C8'—C3'—C2'	121.20 (15)
C8—C3—C2	119.99 (16)	C8'—C3'—C4'	119.05 (16)
C3—C4—H4	119.8	C3'—C4'—H4'	119.8
C5—C4—C3	120.48 (18)	C5'—C4'—C3'	120.41 (17)
C5—C4—H4	119.8	C5'—C4'—H4'	119.8
C4—C5—H5	119.9	C4'—C5'—C6'	120.11 (18)
C6—C5—C4	120.11 (19)	C4'—C5'—H5'	119.9
C6—C5—H5	119.9	C6'—C5'—H5'	119.9
C5—C6—H6	120.0	C5'—C6'—H6'	120.0
C7—C6—C5	119.98 (19)	C7'—C6'—C5'	119.91 (18)
C7—C6—H6	120.0	C7'—C6'—H6'	120.0
C6—C7—C8	120.03 (18)	C6'—C7'—C8'	120.28 (18)
C6—C7—H7	120.0	C6'—C7'—H7'	119.9
C8—C7—H7	120.0	C8'—C7'—H7'	119.9
C3—C8—H8	119.9	C3'—C8'—C7'	120.22 (17)
C7—C8—C3	120.21 (18)	C3'—C8'—H8'	119.9
C7—C8—H8	119.9	C7'—C8'—H8'	119.9
N1—C9—C10	133.02 (15)	N1'—C9'—C10'	133.11 (15)
N1—C9—C14	104.37 (14)	N1'—C9'—C14'	104.09 (14)
C14—C9—C10	122.61 (15)	C14'—C9'—C10'	122.80 (15)
C9—C10—H10	122.1	C9'—C10'—H10'	122.1
C11—C10—C9	115.72 (16)	C11'—C10'—C9'	115.79 (16)
C11—C10—H10	122.1	C11'—C10'—H10'	122.1
C10—C11—C12	122.25 (17)	C10'—C11'—C12'	121.91 (18)
C10—C11—H11	118.9	C10'—C11'—H11'	119.0
C12—C11—H11	118.9	C12'—C11'—H11'	119.0
C11—C12—H12	119.2	C11'—C12'—H12'	119.0
C13—C12—C11	121.64 (17)	C13'—C12'—C11'	122.00 (17)
C13—C12—H12	119.2	C13'—C12'—H12'	119.0
C12—C13—C14	117.06 (16)	C12'—C13'—C14'	116.67 (17)
C12—C13—H13	121.5	C12'—C13'—H13'	121.7
C14—C13—H13	121.5	C14'—C13'—H13'	121.7
N3—C14—C9	108.29 (14)	N3'—C14'—C9'	108.62 (15)
N3—C14—C13	130.99 (16)	N3'—C14'—C13'	130.53 (16)
C9—C14—C13	120.68 (16)	C9'—C14'—C13'	120.83 (16)
C1—C15—H15A	109.2	C1'—C15'—H15C	109.3
C1—C15—H15B	109.2	C1'—C15'—H15D	109.3
C16—C15—C1	111.91 (13)	C16'—C15'—C1'	111.63 (13)
C16—C15—H15A	109.2	C16'—C15'—H15C	109.3
C16—C15—H15B	109.2	C16'—C15'—H15D	109.3
H15A—C15—H15B	107.9	H15C—C15'—H15D	108.0
C17—C16—C15	123.48 (15)	C17'—C16'—C15'	123.46 (15)
C17—C16—C21	115.01 (16)	C17'—C16'—C21'	115.44 (15)
C21—C16—C15	121.47 (15)	C21'—C16'—C15'	121.08 (15)
C16—C17—Cl1	119.61 (13)	C16'—C17'—Cl1'	120.24 (12)
C18—C17—Cl1	117.19 (14)	C18'—C17'—Cl1'	116.96 (14)
C18—C17—C16	123.21 (16)	C18'—C17'—C16'	122.80 (16)
C17—C18—C19	118.98 (18)	C17'—C18'—H18'	120.4
C17—C18—H18	120.5	C19'—C18'—C17'	119.14 (17)
C19—C18—H18	120.5	C19'—C18'—H18'	120.4
C18—C19—H19	119.8	C18'—C19'—H19'	119.7
C20—C19—C18	120.31 (17)	C20'—C19'—C18'	120.56 (16)
C20—C19—H19	119.8	C20'—C19'—H19'	119.7
C19—C20—C21	119.15 (17)	C19'—C20'—C21'	118.90 (16)
C19—C20—H20	120.4	C19'—C20'—H20'	120.6
C21—C20—H20	120.4	C21'—C20'—H20'	120.6
C16—C21—Cl2	118.25 (14)	C20'—C21'—C16'	123.15 (16)
C20—C21—Cl2	118.42 (14)	C20'—C21'—Cl2'	118.11 (14)
C20—C21—C16	123.33 (17)	C16'—C21'—Cl2'	118.74 (13)
			
C1—N1—N2—N3	−176.70 (14)	N2'—N1'—C1'—C2'	45.82 (19)
C9—N1—N2—N3	−0.29 (19)	N2'—N1'—C1'—C15'	−78.65 (17)
N2—N1—C1—C2	−46.4 (2)	C9'—N1'—C1'—C2'	−140.11 (16)
N2—N1—C1—C15	78.61 (18)	C9'—N1'—C1'—C15'	95.42 (18)
C9—N1—C1—C2	137.85 (17)	N2'—N1'—C9'—C10'	−179.53 (17)
C9—N1—C1—C15	−97.12 (18)	N2'—N1'—C9'—C14'	−0.33 (17)
N2—N1—C9—C10	179.28 (18)	C1'—N1'—C9'—C10'	5.9 (3)
N2—N1—C9—C14	0.33 (18)	C1'—N1'—C9'—C14'	−174.94 (15)
C1—N1—C9—C10	−4.6 (3)	N3'—N2'—N1'—C9'	0.62 (18)
C1—N1—C9—C14	176.47 (15)	N3'—N2'—N1'—C1'	175.64 (13)
N1—N2—N3—C14	0.12 (18)	N1'—N2'—N3'—C14'	−0.63 (17)
N2—N3—C14—C9	0.09 (19)	N2'—N3'—C14'—C9'	0.42 (18)
N2—N3—C14—C13	−177.81 (18)	N2'—N3'—C14'—C13'	179.01 (18)
N1—C1—C2—O1	−48.50 (17)	N1'—C1'—C2'—O1'	48.02 (17)
N1—C1—C2—C3	−170.92 (13)	N1'—C1'—C2'—C3'	170.11 (13)
C15—C1—C2—O1	−172.54 (13)	C15'—C1'—C2'—O1'	171.61 (13)
C15—C1—C2—C3	65.04 (17)	C15'—C1'—C2'—C3'	−66.30 (17)
N1—C1—C15—C16	63.47 (17)	N1'—C1'—C15'—C16'	−65.81 (17)
C2—C1—C15—C16	−171.46 (13)	C2'—C1'—C15'—C16'	169.90 (13)
C4—C3—C2—O1	133.27 (17)	O1'—C2'—C3'—C4'	49.0 (2)
C4—C3—C2—C1	−105.93 (19)	O1'—C2'—C3'—C8'	−132.25 (16)
C8—C3—C2—O1	−49.4 (2)	C1'—C2'—C3'—C4'	−71.69 (19)
C8—C3—C2—C1	71.4 (2)	C1'—C2'—C3'—C8'	107.05 (17)
C8—C3—C4—C5	−0.4 (3)	C2'—C3'—C4'—C5'	177.60 (15)
C2—C3—C4—C5	176.99 (18)	C8'—C3'—C4'—C5'	−1.2 (3)
C2—C3—C8—C7	−176.87 (17)	C2'—C3'—C8'—C7'	−177.86 (16)
C4—C3—C8—C7	0.5 (3)	C4'—C3'—C8'—C7'	0.9 (3)
C3—C4—C5—C6	−0.3 (3)	C3'—C4'—C5'—C6'	0.6 (3)
C7—C6—C5—C4	0.8 (3)	C7'—C6'—C5'—C4'	0.3 (3)
C8—C7—C6—C5	−0.6 (3)	C5'—C6'—C7'—C8'	−0.5 (3)
C3—C8—C7—C6	0.0 (3)	C3'—C8'—C7'—C6'	0.0 (3)
N1—C9—C10—C11	−176.93 (18)	N1'—C9'—C10'—C11'	178.29 (18)
C14—C9—C10—C11	1.9 (3)	C14'—C9'—C10'—C11'	−0.8 (3)
N1—C9—C14—N3	−0.25 (18)	N1'—C9'—C14'—N3'	−0.05 (18)
N1—C9—C14—C13	177.90 (16)	N1'—C9'—C14'—C13'	−178.80 (16)
C10—C9—C14—N3	−179.34 (16)	C10'—C9'—C14'—N3'	179.25 (15)
C10—C9—C14—C13	−1.2 (3)	C10'—C9'—C14'—C13'	0.5 (3)
C9—C10—C11—C12	−0.8 (3)	C9'—C10'—C11'—C12'	0.4 (3)
C13—C12—C11—C10	−0.9 (3)	C10'—C11'—C12'—C13'	0.2 (3)
C14—C13—C12—C11	1.6 (3)	C14'—C13'—C12'—C11'	−0.5 (3)
C12—C13—C14—N3	177.08 (18)	N3'—C14'—C13'—C12'	−178.28 (18)
C12—C13—C14—C9	−0.6 (3)	C9'—C14'—C13'—C12'	0.2 (3)
C17—C16—C15—C1	−99.63 (18)	C17'—C16'—C15'—C1'	98.63 (18)
C21—C16—C15—C1	78.26 (19)	C21'—C16'—C15'—C1'	−79.95 (19)
C15—C16—C17—Cl1	−2.1 (2)	C15'—C16'—C17'—Cl1'	1.2 (2)
C15—C16—C17—C18	177.91 (16)	C15'—C16'—C17'—C18'	−178.60 (15)
C21—C16—C17—Cl1	179.90 (12)	C21'—C16'—C17'—Cl1'	179.83 (12)
C21—C16—C17—C18	−0.1 (2)	C21'—C16'—C17'—C18'	0.1 (2)
C15—C16—C21—Cl2	1.3 (2)	C15'—C16'—C21'—C20'	179.40 (16)
C15—C16—C21—C20	−178.87 (16)	C17'—C16'—C21'—C20'	0.7 (2)
C17—C16—C21—Cl2	179.38 (12)	C15'—C16'—C21'—Cl2'	−0.5 (2)
C17—C16—C21—C20	−0.8 (3)	C17'—C16'—C21'—Cl2'	−179.16 (12)
Cl1—C17—C18—C19	−179.39 (14)	Cl1'—C17'—C18'—C19'	179.82 (13)
C16—C17—C18—C19	0.6 (3)	C16'—C17'—C18'—C19'	−0.4 (3)
C17—C18—C19—C20	−0.2 (3)	C17'—C18'—C19'—C20'	0.0 (3)
C21—C20—C19—C18	−0.6 (3)	C21'—C20'—C19'—C18'	0.7 (3)
Cl2—C21—C20—C19	−179.01 (14)	C19'—C20'—C21'—Cl2'	178.75 (14)
C16—C21—C20—C19	1.2 (3)	C19'—C20'—C21'—C16'	−1.1 (3)

### Hydrogen-bond geometry (Å, °)

**Table:**

*D*—H···*A*	*D*—H	H···*A*	*D*···*A*	*D*—H···*A*
O1—H1A···N2	0.82	2.56	3.1254 (19)	127
O1'—H1B···N2'	0.82	2.52	3.0881 (19)	128
C19—H19···N3^i^	0.93	2.54	3.420 (2)	158
C19'—H19'···N3'^ii^	0.93	2.53	3.410 (2)	159

Symmetry codes: (i) *x*+1, *y*, *z*; (ii) *x*, *y*+1, *z*.
